# Comparison of Topical Anesthetics for Radiofrequency Ablation of Achrocordons: Eutectic Mixture of Lignocaine/Prilocaine versus Lidocaine/Tetracaine

**DOI:** 10.1155/2014/743027

**Published:** 2014-01-29

**Authors:** Pratik Gahalaut, Nitin Mishra, Sandhya Chauhan, Madhur Kant Rastogi

**Affiliations:** ^1^Department of Dermatology, Venereology and Leprosy, Shri Ram Murti Smarak Institute of Medical Sciences, Nainital Road, Bareilly 243001, India; ^2^Department of Pediatrics, Shri Ram Murti Smarak Institute of Medical Sciences, Nainital Road, Bareilly 243001, India

## Abstract

*Introduction*. Topical application of local anesthetics is currently considered to be the easiest, most effective, and convenient way for treatment of patients who may be undergoing superficial dermatosurgical procedures. *Materials and Methods*. This study compares the anesthetic potential of 2.5% lidocaine and 2.5% prilocaine topical cream with 7% lignocaine and 7% tetracaine combination cream for radio ablative dermatosurgery when applied, under occlusion, for 30 minutes. 40 subjects of achrocordons were enrolled in this split-side randomized trial. *Result*. The pain severity experienced by subjects in terms of visual analogue scale score was significantly lesser for lignocaine/tetracaine combination cream as compared to lidocaine/prilocaine combination. *Conclusion*. This small study proves the efficacy of lidocaine/tetracaine combination as a topical anesthetic cream when applied for a short time interval of 30 minutes. This will help a dermatosurgeon to perform various dermatological procedures in a better and efficient manner with a shorter waiting period for analgesia to set in.

## 1. Introduction

Radiofrequency ablation (radiosurgery, high frequency electrosurgery) is a dermatosurgical procedure that aims at the surgical management of benign and malignant skin conditions by using various forms of alternating current at ultrahigh frequency (500–4000 kHz) [1]. Radiofrequency surgery has gained importance in the recent years as it has distinct advantages, like less bleeding, cutting as well as coagulation ability, minimal tissue trauma, faster healing, and good aesthetic results [2]. Nowadays most of the dermatologists prefer using radiosurgery in place of electrosurgery units. Dermatological procedures may be associated with pain and discomfort. For some patients, the procedural pain, associated stress, and anxiety represent a significant clinical concern [3]. This can be alleviated to a great extent by using a local anesthetic.

Local anesthesia can be administered by injection or through the use of topical creams [4]. Traditionally, intradermal injection of lidocaine (with or without the use of epinephrine) has been the method of choice for induction of local anesthesia [5, 6]. Local anesthetic, that is, lignocaine, is administered before most radio-surgical techniques [1, 2]. However, injectable anesthetics are often painful, are difficult to use in “needlephobic” patients, and may result in anatomic distortions which is unacceptable in cosmetic procedures [4]. Many topical anesthetic creams have become available over the last few years and they claim to provide a long-lasting anesthetic effect after topical use on the skin [7]. The potential benefits for patch and cream delivery systems over injection include increased safety, convenience of use, ease of disposal, reduced drug loading, and no tissue dissention [8]. Topical application of local anesthetics is currently considered to be the easiest, most effective, and convenient way for treatment of patients who may be undergoing superficial dermal procedures. Application of topical anesthetics before or in place of injection of local anesthetic can help to relieve anxiety [9]. The most commonly used dermal analgesics are lidocaine, tetracaine, prilocaine, or combinations thereof [10].

The most common topical anesthetic and often regarded as the gold standard by which other topical anesthetics are compared is EMLA cream (Astra Pharmaceuticals, Westborough, MA, USA) [7, 11]. EMLA cream is an emulsion in which the oil phase is a eutectic mixture of 2.5% lidocaine and 2.5% prilocaine (LP) [1, 2]. EMLA cream is a recommended alternative topical anesthetic for radiofrequency ablation if applied under occlusion to skin at least 1 h before the procedure [1].

Recently the Food and Drug Administration (FDA), USA, has approved a stable compounded mixture of 7% lidocaine and 7% tetracaine (LT) cream. The LT formulation has the highest approved concentrations of lidocaine and tetracaine that has also proven to be safe and effective in producing adequate local dermal anesthesia for dermatological procedures if placed on intact skin for a time interval as short as 20 minutes only [4, 9]. Topical anesthesia with a waiting time of 30 minutes would be more viable and convenient to use in dermatology procedures compared to the one requiring a downtime of at least 1 hour.

In the past, 7% lignocaine and 7% tetracaine (LT) combination has been compared with 2.5% lignocaine and 2.5% prilocaine (LP) combination anesthetic creams for vascular access and laser resurfacing [3, 11]. However studies comparing the anesthetic potential of these two combinations for superficial dermatosurgical procedures after only 30-minute application are lacking. Hence this study was designed to compare the patient's acceptability and efficacy of LT and LP combination anesthetic cream after 30-minute application for radio ablation of achrocordon (skin tag) from the intact skin of neck. In our country both LT and LP combinations are freely available under different brand names and all these brands are approved by national drug controlling authorities. However, unlikely worldwide, the LT combination which is available in our country has to be applied under occlusion. This was a blessing in disguise as it made the blinding of cream application easier and nondifferentiable.

## 2. Material and Methods

This prospective, randomized trial was designed to compare the effectiveness of the lidocaine/tetracaine combination cream with that of lidocaine/prilocaine combination cream when administered for 30 minutes, under occlusion, to provide cutaneous anesthesia for radio ablation of achrocordons. A written informed consent was taken from all volunteers before their participation and study was approved by institutional ethics committee. This study was conducted in the Department of Dermatology of Shri Ram Murti Smarak Institute of Medical Sciences, Bareilly (UP), India.

Inclusion criteria were adults, who were 18 to 60 years of age, having achrocordons in axillae bilaterally as per clinical examination. Exclusion criteria were pregnancy; lactation; use of any other topical or systemic analgesic; history of any antidepressant or antiepileptic medications in the last 3 months; patients with known allergy to any anesthetic in the past; damaged, denuded, or broken skin at the chosen site of anesthetic cream application.

### 2.1. Study Design

For inclusion in this study, subjects were required to have achrocordons in both axillae. Area measuring roughly 15 mm × 20 mm size was chosen in either axilla for occlusion by study cream and all the achrocordons lying in this chosen area were removed using a Megasurge gold radiosurgery unit (Derma-India Company, Chennai, India) using cut mode at the power setting of 3 as per recommendations of IADVL taskforce on radiosurgery [2]. Care was taken that the current intensity was constant throughout the process. If the subject had more achrocordons in other areas, then subsequent achrocordons were removed in another sitting based on the choice expressed by him/her regarding the type of anesthesia. LP cream was applied on the chosen site in axilla on one side only and LT combination was applied on the symmetrical area of contralateral axilla in every subject. The right or left side was chosen for the respective cream application based on a computer generated randomization table. Subjects and the sole operating investigator were blind to this randomization. Study agents were applied by a nonparticipating nurse in a layer approximately 1-2 mms thick and covered with a transparent polyurethane dressing (Tegaderm, 3M Pharmaceuticals, Neuss, Germany) on both sides. After the 30-minute application, the nonparticipating nurse removed the study creams from both axillae with the help of a wet saline cotton gauze piece. Subsequently the investigator removed 2–5 achrocordons from the chosen area and the subject was asked about any discomfort. If desired, subject was given the choice of an injectable anesthetic for further radio ablation. If not, then all the lesions were removed from the chosen area. Immediately after this, patient was asked to rate the severity of the discomfort experienced using the visual analogue scale. The whole process took 5–7 minutes on average. The whole exercise was repeated on the other side immediately thereafter. The operating investigator started from the right side always.

### 2.2. Assessment of Pain

Primary efficacy outcomes were measured as pain severity scores as recorded by the patients and patient response in the form of yes/no regarding adequate pain relief during the procedure. Pain severity was assessed with the help of 100 mm vertical VAS (visual analogue scale) with the end points of 0 rated as no pain and 100 as intolerable pain [12]. An independent observer also evaluated the subject's pain intensity during the procedure using a four-point categorical scale as 0 to 3 (0 representing no pain, 1 mild, 2 moderate, and 3 severe pain). Secondary efficacy outcomes were measured as the proportions of subjects having complete anesthesia at the site of anesthetic cream application after 30-minute interval and the proportion of subjects willing to reuse the particular study cream combination for anesthesia in the future.

### 2.3. Statistical Analysis

The two-sided Fisher exact test was used for comparing proportions and the two-sided Wilcoxon signed-rank test was used for comparing pain scores by means of Graph Pad software 6.0 which is available freely on the Internet site http://www.graphpad.com. Test results with *P* values <0.05 were considered significant.

## 3. Results

Forty subjects (27 males and 13 females; mean age 44 years, range 28–59 years) were enrolled in this study. Three subjects complained of intense itching or burning sensation at the site of test drugs application within 15 minutes after applying anesthetic creams. These 3 subjects were withdrawn from the study and subsequently 3 more subjects were enrolled to complete the study. Since this is a split-side study, there was no statistical difference in age, sex, and/or site of lesions.

### 3.1. Efficacy

**Figure 1:**
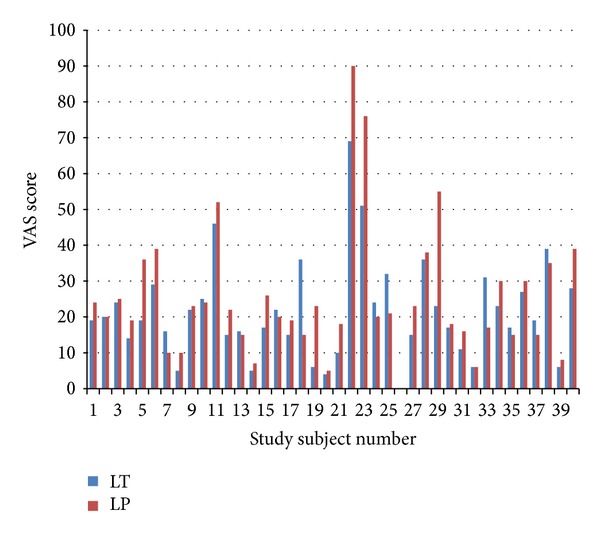
Pain scores after 30 minutes of anesthetic cream application (*P* = 0.0174).

In primary efficacy outcome, LT combination cream showed significantly better anesthesia (Figure 1). Subjects reported significantly lesser VAS score for LT compared to LP combination as per Wilcoxon signed-ranks test (*W* = 312, *P* = 0.0174). The majority of patients (more than 2/3rd) reported adequate pain relief with both the combinations. All the patients tolerated the procedure well and declined the choice of using any other injectable anesthetic for both LT and LP creams. Though more subjects reported adequate pain relief with LT (75%) compared to LP (67.5%), there was no statistically significant difference in the proportions of subjects who reported adequate pain relief between the two combinations (*P* = 0.6219).

In the secondary efficacy outcome, significantly more subjects were willing to reuse LT combination (82.5%) compared to LP combination (60%) in the future (*P* = 0.0340). Only one patient each in either study combination cream reported complete anesthesia and this difference was not significant (*P* = 1.000).

Independent observer's ratings were comparable and showed no significant difference for both the study creams. Observer ratings for LT and LP creams, respectively, were 11/40 versus 11/40 for no pain, 21/40 versus 20/40 for mild pain, 7/40 versus 8/40 for moderate pain, and 1/40 versus 1/40 for severe pain.

### 3.2. Adverse Effects

More patients experienced adverse effects in the form of erythema, edema, or hyperpigmentation at the site of LT cream (7/40) compared to LP cream (3/40). However, this was not statistically significant in the intention to treat analysis (*P* = 0.5179). Blanching occurred at the site of anesthetic cream application only with LP creams.

## 4. Discussion

An ideal topical anesthetic agent would be easy to apply, show high clinical effectiveness over a short time period, exert its effect on intact skin without systemic effects, and cause nominal pain or discomfort during treatment with minimal to no side effects [10, 13, 14]. Topical anesthetics must be able to penetrate the relatively impermeable barrier of the stratum corneum and have minimal systemic absorption [10, 14]. Eutectic mixtures allow individual anesthetic compounds, which are normally in the solid state at room temperature, to be combined as liquids. Eutectic mixtures permit higher concentrations of anesthetic to be used safely and facilitate application to the skin [9].

We found a significant difference in the pain severity experienced by subjects on comparing VAS scores for LT and LP creams. The subjects reported significantly lesser VAS scores for LT cream compared to LP cream after application for 30 minutes. In other words, LT creams provided better analgesia after 30-minute application under occlusion. In the past, in comparison with EMLA (LP combination) cream, Rapydan (LT combination) offers superior analgesia [3]. Lidocaine/tetracaine cream has been compared to EMLA for ablative CO_2_ laser skin resurfacing. Patients who received LT cream had lower pain scores compared to those who received EMLA cream [11]. Chen et al. reported that a 30-minute application of LT formulation in peel form would be adequate for less painful and superficial procedures such as PDL therapy [15].

For superficial dermatosurgical procedures, topical anesthetic with an onset of action within 30 minutes is desirable [16]. Though the present trial compared the analgesic effect of the two anesthetic combinations and not their time of onset, the difference in pain severity score may be due to the faster onset of action for LT combination compared to LP study cream. Recommended application time for LT combination ranges from 30 to 60 minutes.

LT cream Pliaglis (Galderma Laboratories, Texas, USA) is the first FDA-approved stable compounded mixture of 7% lidocaine and 7% tetracaine cream [11]. Pliaglis has a novel vehicle which functions as a self-occluding cream—the product is applied as a cream that dries to a pliable membrane upon exposure to air [14, 17]. No difference in analgesic effect for this formulation has been seen for 30- and 60-minute application periods [14]. S-Caine patch is a 1 : 1 eutectic mixture of 7% lidocaine and 7% tetracaine combination and utilizes controlled heating to reportedly enhance the rate of anesthetic delivery into dermis. Clinical studies have demonstrated that 30-minute administration of S-Caine patch is efficacious in relieving pain associated with shave biopsies, venipuncture, and superficial dermatological procedures [9, 18]. The mean depth of analgesia for S-Caine patch has been measured to be at least 6.8 mm [9]. For Rapydan, a medicated patch containing a mixture of 7% lidocaine and 7% tetracaine with a control heat assisted drug delivery system, the average depth of topical anesthesia is reported to be greater than 3.6 mm at a time interval of 30 minutes after application [3]. The heated lidocaine/tetracaine patch is well tolerated, and it provides favourable depth and duration of anesthesia for minor dermatological procedures after a 30-min application [19].

In our study also LT combination anesthetic cream provided adequate topical anesthesia for radio ablation of achrocordons after application, under occlusion, for 30 minutes. It is noteworthy that LT combination in our study was applied under occlusion as per the recommendation of the manufacturer. The significant higher VAS pain severity scores with LP cream in our study may be due to the shorter 30-minute application time period for study creams in the present study.

For EMLA (LP combination), analgesia is achieved till a depth of 3 mm after 60 minutes of application, and a maximum dermal depth of 5 mm is reached after 120 minutes [9]. EMLA cream requires 40–60 min of application under occlusion for adequate analgesic effect [3, 14]. EMLA does not provide adequate anesthesia at peripheral skin margins [14]. The average time of application in order to achieve adequate anesthesia typically exceeds 60 minutes, limiting the practical use of this combination in a busy clinical practice [4].

A recent meta-analysis states that there was not much difference in efficacy and safety between subgroups of the lidocaine/tetracaine medicated patch and peel for dermal anesthesia for intact skin [10]. Further Kim et al. concluded that use of lidocaine/tetracaine combination was judged to be safe to use [10].

In the present study, both the study combinations of anesthetic creams provided adequate pain relief in more than two-thirds of subjects after 30 minutes of initial application. There was no significant difference in the proportion of subjects reporting adequate pain relief in spite of significant difference in pain severity score experienced by these subjects for LT or LP cream application. This might be because achrocordons are epidermal tumour and hence deeper dermal anesthesia may not be required for them. The extent of anesthesia (whether in terms of reduction of pain or absence of any sensation) and the depth of anesthesia (in millimetres) induced by percutaneously administered anesthetics depend on the duration of the application [16]. Optimal application time for S-Caine peel varies with severity and depth of the procedure [15]. Previous reports have demonstrated that the onset of analgesia on face skin was less than 25 minutes after EMLA application under occlusive dressing [20]. At the same time Singh et al. reported that 75% of patients experienced mild pain after 30 minutes of EMLA application before radio ablation of verrucae [21].

In the present study, more subjects preferred LT combination compared to LP, for reuse. This may be a reflection of the better analgesic effect of this combination after 30-minute application time. The results of our study are consistent with past studies of LT combination [3, 6, 22–24].

In this study, both the LT and LP combinations were well tolerated and no significant difference was seen with respect to side effects. Erythema, blanching, and edema are known side effects of LT combination [3]. The potential for systemic absorption of tetracaine and lidocaine through intact skin is insignificant because blood concentrations of tetracaine and lidocaine were reported to be below the lower limit of quantization [10].

### 4.1. Limitations of This Study

The smaller number of subjects in this study may be the reason for nonsignificant difference in the proportion of subjects reporting adequate pain relief with either LT or LP combination anesthetic creams. We did not factor any bias while determining sample size for “an order of injection” effect which states that the initial experience of noxious stimulus lowers the pain perception threshold for subsequent stimuli [16]. However the study investigator always started the procedure from the right side so that this confounding bias may be minimized. Though both the subjects and operating surgeon were blinded, the blanching or erythema which occurs at the site of study cream application might have provided a clue to operating dermatosurgeon regarding the type of combination applied at that particular site/side. Again this would not have affected the final result because the scoring was done by either the subjects or an independent observer.

## 5. Conclusion

The most significant disadvantage with the use of topical anesthetic creams is the time taken for the local anesthetic effect [7]. Our small study has demonstrated that, for superficial radio ablation of achrocordons, LT combination anesthetic cream provides better analgesia compared to LP combination after application for 30 minutes under occlusion. Hence both, the operating dermatosurgeon and the patient, will benefit immensely from the faster acting and effective lidocaine/tetracaine combination anesthetic cream. For superficial dermatological procedures like radio ablation, the combination of lidocaine/tetracaine is the best topical anesthetic if not the ideal, in the present scenario. It provides adequate pain relief, in lesser time period, with negligible side effects, if used cautiously. However, larger randomized controlled trials are required to conclusively prove this hypothesis for deeper invasive procedures.
